# Peripherally increased artemin is a key regulator of TRPA1/V1 expression in primary afferent neurons

**DOI:** 10.1186/s12990-015-0004-7

**Published:** 2015-03-08

**Authors:** Yasuko Ikeda-Miyagawa, Kimiko Kobayashi, Hiroki Yamanaka, Masamichi Okubo, Shenglan Wang, Yi Dai, Hideshi Yagi, Munetaka Hirose, Koichi Noguchi

**Affiliations:** Department of Anatomy and Neuroscience, Hyogo College of Medicine, 1-1 Mukogawa-cho, Nishinomiya, Hyogo 663-8501 Japan; Department of Anesthesiology, Hyogo College of Medicine, 1-1 Mukogawa-cho, Nishinomiya, Hyogo 663-8501 Japan; Department of Pharmacy, School of Pharmacy, Hyogo University of Health Sciences, Kobe, Hyogo 650-8530 Japan

## Abstract

**Background:**

Artemin, a member of the glial cell line-derived neurotrophic factor family, is known to have a variety of neuronal functions, and has been the subject of attention because it has interesting effects, including bi-directional results in modulation in neuropathic and inflammatory pain. It has been shown that the overexpression of artemin is associated with an increase in the expression of TRP family channels in primary afferents and subsequent hyperalgesia, and an increase in neuronal activity. The purpose of this study was to examine the peripheral synthesis of artemin in inflammatory and neuropathic pain models, and to demonstrate the effects of long-term or repeated application of artemin *in vivo* on pain behaviors and on the expression of TRP family channels. Further, the regulatory mechanisms of artemin on TRPV1/A1 were examined using cultured DRG neurons.

**Results:**

We have demonstrated that artemin is locally elevated in skin over long periods of time, that artemin signals significantly increase in deep layers of the epidermis, and also that it is distributed over a broad area of the dermis. In contrast, NGF showed transient increases after peripheral inflammation. It was confirmed that the co-localization of TRPV1/A1 and GFRα3 was higher than that between TRPV1/A1 and TrkA. In the peripheral sciatic nerve trunk, the synthesis of artemin was found by RT-PCR and *in situ* hybridization to increase at a site distal to a nerve injury. We demonstrated that *in vivo* repeated artemin injections into the periphery changed the gene expression of TRPV1/A1 in DRG neurons without affecting GFRα3 expression. Repeated artemin injections also induced mechanical and heat hyperalgesia. Using primary cultured DRG neurons, we found that artemin application significantly increased TRPV1/A1 expression and Ca^2+^ influx. Artemin-induced p38 MAPK pathway regulated the TRPV1 channel expression, however TRPA1 upregulation by artemin is not mediated through p38 MAPK.

**Conclusions:**

These data indicate the important roles of peripherally-derived artemin on the regulation of TRPV1/A1 in DRG neurons in pathological conditions such as inflammatory and neuropathic pain.

## Background

Artemin, a member of the glial cell line-derived neurotrophic factor family, is involved in a variety of neuronal functions such as development, regeneration and regulation of gene expression and neural activity. Artemin binds to the GFRα3/RET receptor complex and then activates several intracellular signaling pathways [1]. Among several neurotrophic factors working in nociceptive pathways, artemin has been the subject of attention due to its unique characteristics that were recently reported. One important characteristic is that the receptor of artemin, GFRα3, is selectively expressed in adults to a subpopulation of nociceptive sensory and sympathetic neurons. This is also colocalized with the transient receptor potential (TRP) ion channel family proteins [2,3].

TRPV1/TRPA1 is a member of TRP family of cation channels [4] and is expressed by a subset of small-sized DRG or trigeminal ganglia neurons [5-7]. In addition to the established effect of TRPV1 on noxious heat transduction, there have been many reports on the role of TRPA1; various kinds of noxious compounds activate TRPA1 through covalent modification of cysteines [5,8-13], and TRPA1 is also activated by an endogenous aldehyde, 4-hydroxynonenal, bradykinin, intracellular pH and CO_2_ [8,14-16]. Several papers have indicated that TRPA1 is an important component of the transduction machinery through which noxious irritants and endogenous proalgesic molecules depolarize nociceptors to elicit inflammatory pain [17,18]. Therefore, there has been interest in the study of TRPA1 as it is crucial in pathological pain transduction, and the regulatory mechanisms of TRPA1 in persistent pain condition require further studies.

It has been reported that artemin has bi-directional results in the modulation in neuropathic and inflammatory pain. For example, administration of artemin prevented changes in the nociceptive pathway after peripheral nerve injury [19-22], and reversed nerve injury-induced pain behaviors [20]. Conversely, artemin is reported to have a pronociceptive role in peripheral inflammation or nerve injury. The overexpression of artemin increased the expression of TRP channels in primary afferents and subsequent hyperalgesia [23], and artemin administration induced a significant potentiation of TRPV1 function *in vitro* and thermal hyperalgesia *in vivo* [24]. Further, artemin may have different effects on TRPV1 and TRPA1, namely an inhibitory effect on TRPA1 activity [25], and a pronociceptive role on TRPV1 function [24]. Therefore, we intended to examine in detail the long-term effects of artemin on pain behaviors and on the expression of TRP family channels using *in vivo* and cultured DRG neurons. We observed an increase in artemin expression in the epidermis of inflamed skin and also peripheral nerves distal to a nerve injury. Increased artemin at peripheral sites was shown to affect the gene expression of TRPA1/V1 in primary afferents both *in vivo* and *in vitro*. All findings in the present study suggest the pivotal role of peripheral tissue-derived artemin in the regulation of TRPA1/V1 expression in primary afferents.

## Results

### Artemin increased locally in skin over long periods of time, whereas NGF showed only transient increases after peripheral inflammation

**Figure 1:**
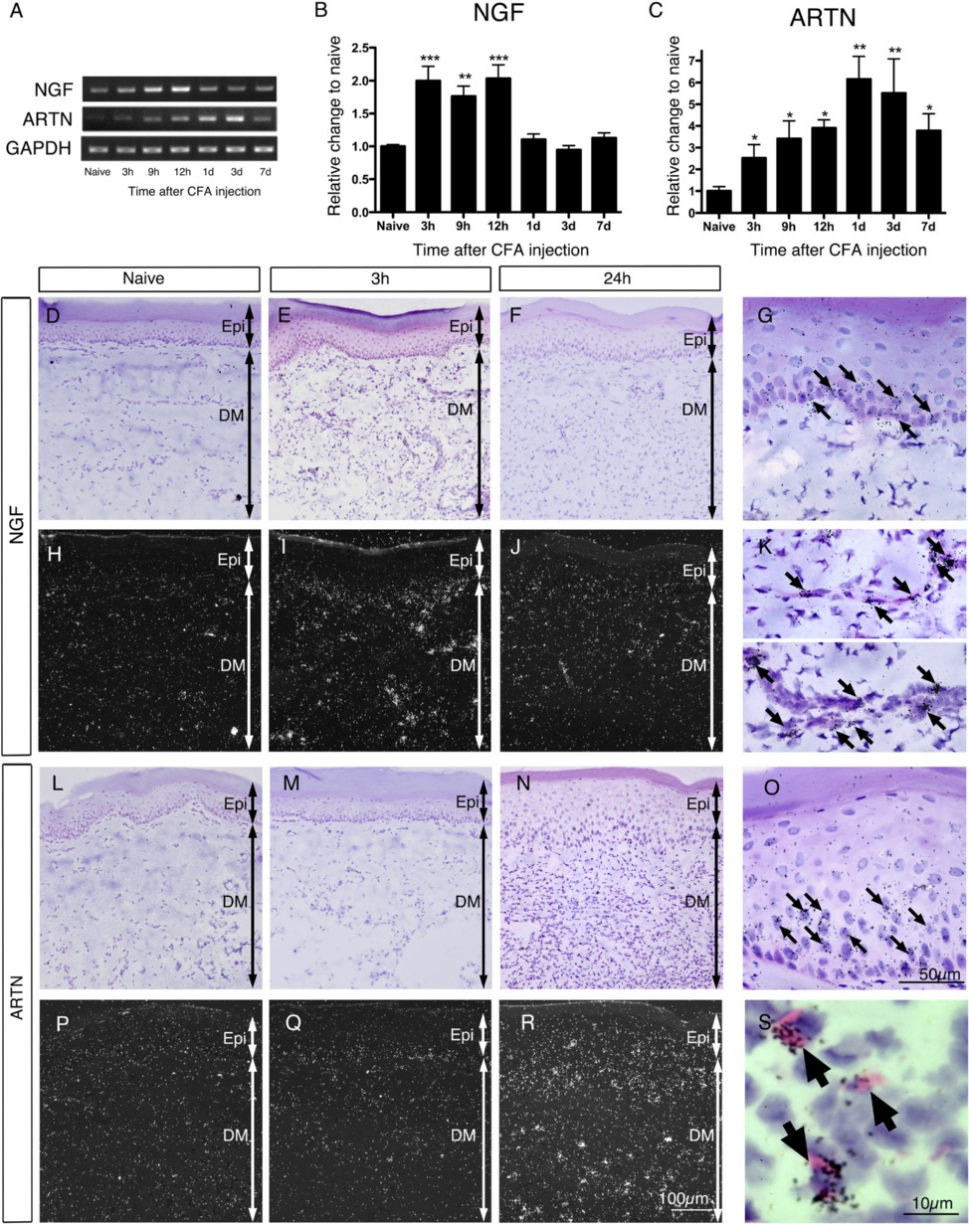
**Artemin increases locally in skin over long periods of time, whereas NGF shows a transient increase after peripheral inflammation. A**-**C**: The NGF mRNA level almost doubled compared to the naïve level after 3 hours and sustained the same level until 12 hours, but returned to the normal level by 24 hours post CFA injection. In contrast, the artemin (ARTN) mRNA level showed a dramatic increase, more than doubling in 3 hours, and remained elevated until 7 days after injection (n = 4, mean ± SEM, ***p < 0.001, **p < 0.01, *p < 0.05, compared with naïve). **D**-**S**: ISHH for plantar skin revealed the source of NGF and artemin. NGF mRNA was expressed 3 hours after CFA injection **(E, I)** and it was expressed from the keratinocytes in the epidermis (**G**, arrows) and vascular smooth muscles in the dermis (**K**, arrows). Artemin mRNA was observed over a broad area of the epidermis 24 hours after CFA injection as seen in the dark-field photomicrograph (**N** and **R**). The artemin-generating cells were presumably keratinocytes in the epidermis (**O**, arrows) and eosin-labeled immune cells in the dermis according to the magnified bright-field microphotographs (**S**, arrows). Epi: epidermis, DM: dermis. Bars: 100 μm **(D-F, H-J, L-N, P-R)**, 50 μm **(G, K, O)**, 10 μm **(S)**.

In order to examine the local synthesis of growth factors after tissue inflammation, we injected complete Freund’s adjuvant (CFA) into rats’ plantar surfaces and measured the changes in the mRNA of nerve growth factor (NGF) and artemin in the skin using RT-PCR (Figure 1A). The NGF mRNA level almost doubled compared to the naive level after 3 hours, and sustained the same level until 12 hours, but returned to normal by 24 hours post-injection (Figure 1B). In contrast, artemin mRNA levels showed dramatic increases, more than doubling 3 hours after injection, and remaining elevated until 7 days after injection, where the peak was at 1 day with a six-fold increase (p < 0.05 versus naive, Figure 1C).

To identify the source of NGF and artemin synthesis in inflamed tissue, we used *in situ* hybridization histochemistry (ISHH) for the plantar skin (Figure 1D-S). Consistent with the RT-PCR data, an increase in NGF mRNA was detected 3 hours after CFA injection (Figure 1E, I). Dark-field photomicrographs for NGF showed that clusters of silver grains increased mainly in the dermis and hypodermis (Figure 1H-I), and it was presumed that the NGF was expressed mainly in keratinocytes in the epidermis (Figure 1G) and vascular smooth muscle (Figure 1K). NGF mRNA was faintly expressed one day after CFA injection (Figure 1F, J). By contrast, the increase in artemin mRNA was mild compared to that of NGF. As seen in the dark-field photomicrograph one day after CFA injection, a high intensity of silver grains was observed over a broad area of the epidermis and dermis (Figure 1P-R). The silver grains in the epidermis were increased in density and scattered through deep layers of the epidermis. Signals were also detected in cells located in the dermis and hypodermis. The artemin-generating cells include keratinocytes in the epidermis (Figure 1O), and eosin-labeled immune cells in the dermis (Figure 1S) according to the magnified bright-field microphotographs.

### Artemin increases in the peripheral sciatic nerve following Wallerian degeneration after nerve injury

**Figure 2:**
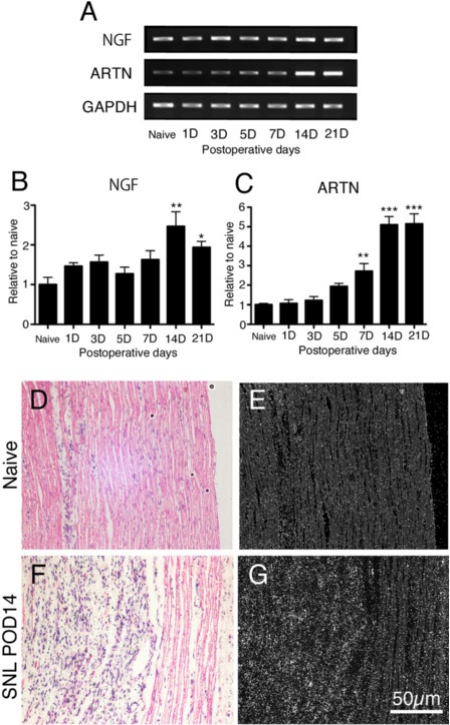
**Artemin increases in peripheral sciatic nerve following Wallerian degeneration after spinal nerve ligation. A**-**C**: NGF and artemin mRNA levels in the ipsilateral sciatic nerve were measured by PT-PCR. The NGF mRNA level increased at 14 and 21 days after SNL (**B**, n = 4, mean ± SEM, **p < 0.01, *p < 0.05 compared with naïve). Artemin mRNA levels gradually increased and continued until 21 days after SNL (**C**, n = 4, mean ± SEM, **p < 0.001, ***p < 0.0001, compared with naïve). **D**-**G**: The area of the L5 spinal nerve in the ipsilateral sciatic nerve showed Wallerian degeneration in the bright-field microphotographs 14 days after ligation and ISHH revealed that artemin mRNA was in the same area as the Wallerian degeneration. Bars: 50 μm **(D-G)** POD; post operative day.

We examined the expression of NGF and artemin in peripheral nerves after nerve injury. After the spinal nerve ligation (SNL), NGF and artemin mRNA showed different patterns of increase at a point distal to the nerve injury. NGF showed a small increase that significantly increased at 14 and 21d after nerve injury. In contrast, artemin mRNA showed a gradual and large increase, which significantly increased from 1–3 weeks after nerve injury (Figure 2A-C). To determine the area expressing increased artemin mRNA, we performed ISHH on the sciatic nerve after SNL. We found that large portion of the sciatic nerve had Wallerian degeneration, and these degenerated areas coincided with areas expressing increased artemin mRNA at 14d after nerve injury (Figure 2D-G). Collectively, artemin showed a longer increase in synthesis at peripheral sites in both inflammation and nerve injury models compared to NGF.

### Repeated artemin injections changed the gene expression of TRPV1 and TRPA1 in DRG neurons

**Figure 3:**
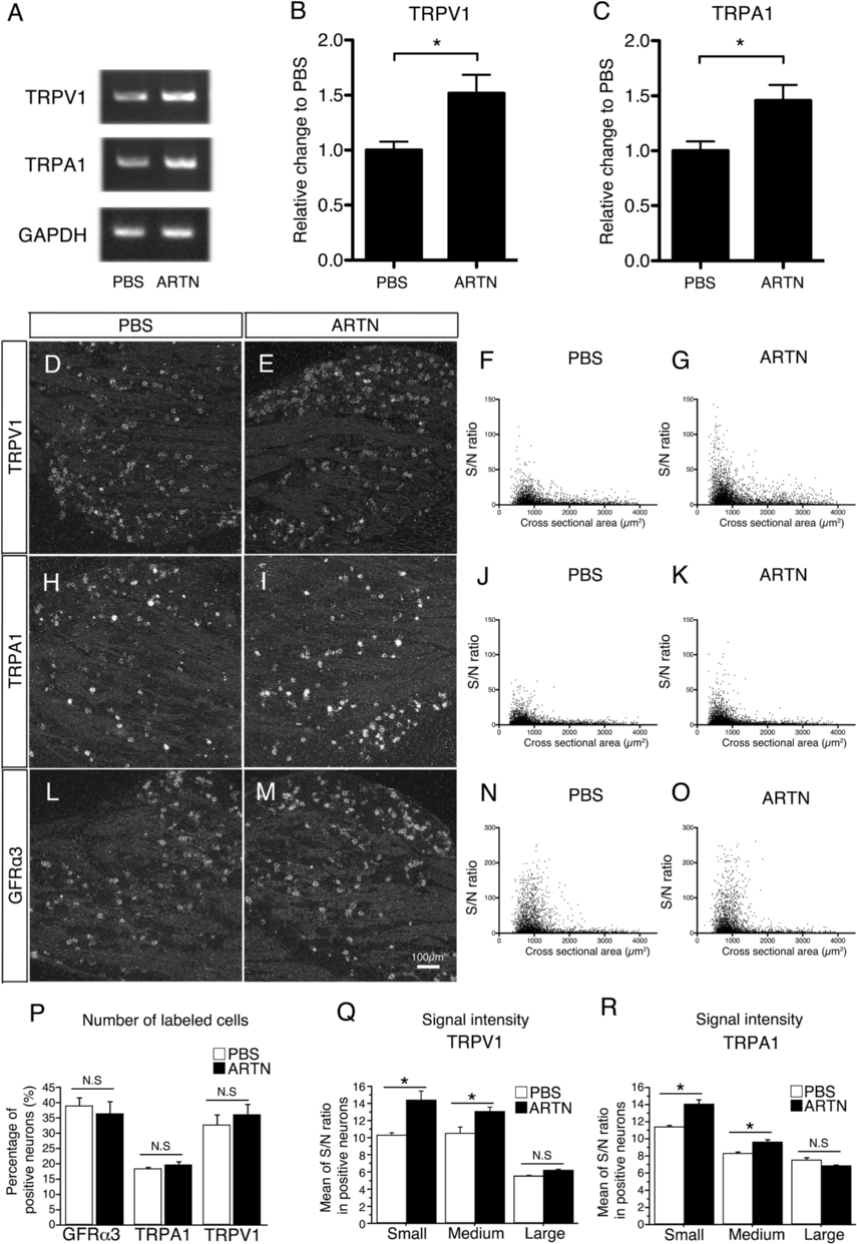
**Repeated artemin plantar injections changed the gene expressions of TRPV1 and TRPA1 in DRG neurons. A**-**C**: Artemin was injected into the planter surface once a day for 5 days (20 μg/ml, 100 μl/injection, PBS control, n = 8 each groups) and TRPV1 and TRPA1 mRNA levels in DRG were both increased significantly compared with control (mean ± SEM, *p < 0.05). **D**-**O**, ISHH and the scatterplots of TRPV1, TRPA1 and GFRα3 mRNAs in DRG neurons after PBS **(D, F, H, J, L, N)** or artemin injection **(E, G, I, K, M, O)**. Each DRG neuron was plotted with the signal-to-noise (S/N) ratio and its cross-sectional area. The number of TRPV1/TRPA1 mRNA expressing neurons is quantified **(P)** and the mean S/N ratio in positive neurons was shown in **Q** and **R**. The total number of TRPV1 or TRPA1 mRNA-labeled neurons was not significantly increased **(P)**, while the signal intensity of both mRNAs after artemin injection were clearly increased after repeated artemin injection in small and medium-sized DRG neurons compared to PBS control **(Q, R)** (mean ± SEM, *p < 0.05). Repeated artemin injection did not change the profiles of GFRα3, which is a receptor of artemin. Bars: 100 μm **(D, E, H, I, L, M)**.

NGF is believed to be an important modulator of gene expression in primary afferents. The results presented in Figures 1 and 2 suggested that the increase in artemin has a longer time course after peripheral inflammation and nerve injury compared to NGF. A previous study indicated that overexpression of artemin caused increased TRPV1 in the DRG *in vivo* [2]. Therefore, we examined the changes in gene expression of TRPV1 and TRPA1 in DRG neurons after repeated artemin injections, using RT-PCR and ISHH of DRG neurons. Five days of injections of artemin (20 μg/ml, 100 μl/injection) into the planter surface significantly induced TRPV1 and TRPA1 mRNA in the DRG measured by RT-PCR (Figure 3A-C). Next, we examined the mRNA changes in the DRG neurons using ISHH and measured the signal-to-noise (S/N) ratio in each DRG neuron plotted on scatterplot diagrams (Figure 3D-O). The number of TRPV1 mRNA-expressing neurons was not significantly increased after repeated artemin injections (Figure 3D-G, P); however, the signal intensity was increased in small and medium-sized DRG neurons (Figure 3Q), and these increases were statistically significant. Next, the TRPA1 mRNA was examined in DRG neurons after repeated artemin injection. The signal intensity of TRPA1 mRNA increased in small and medium-sized DRG neurons after artemin injection compared to controls (Figure 3H-K, R), but the total number of TRPA1-expressing neurons did not increase compared to the PBS control (Figure 3P). This pattern of increase of TRPA1 mRNA was similar to that in TRPV1. We also investigated GFRα3 mRNA and found that repeated artemin injection did not change the expression of GFRα3 (Figure 3L-P). These results confirmed that persistent artemin administration in the peripheral tissue significantly increased the gene expression of TRPA1 and TRPV1 in small to medium-sized DRG neurons without changing their receptor profiles.

### The co-localization of TRPV1/A1 and GFRα3 was higher than that of TRPV1/A1 and TrkA

**Figure 4:**
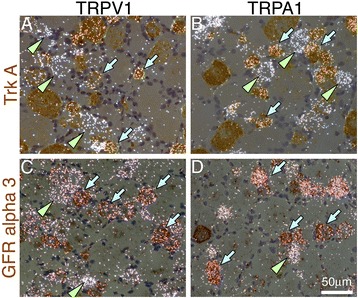
**The co-localization of TRPV1/A1 and GFRα3 was higher than that of TRPV1/A1 and TrkA. A**-**D**: Colocalization of TRPV1 or TRPA1 mRNA (clusters of solver grains; triangle heads) and TrkA (**A** and **B**) or GFRα3 (**C** and **D**) mRNA (brown cells) using double ISHH with ^35^S-labeled antisense TRPV1 or TRPA1 probes combined with DIG-labeled TrkA or GFRα3 probe. The brightfield and darkfield photographs are merged. Arrows indicate examples of double-labeled neurons. Bars: 50 μm **(A-D)**.

Next, we compared the receptor of artemin in primary afferents with that of NGF. We studied the co-localization of TRPA1 or TRPV1 with Trk A or GFRα3 in DRG neurons (Figure 4). We found that 43.2 ± 3.9% of the TRPA1 positive neurons and 47.5 ± 6.2% of the TRPV1 positive neurons expressed TrkA. In the case of the artemin receptor, 68.5 ± 5.4% of TRPV1 positive neurons overlapped with GFRα3 mRNA, and 84.1 ± 2.6% of TRPA1 expressing neurons were also labeled with GFRα3 mRNA. These results indicated that TRPA1/V1-expressing primary afferent neurons contained the artemin receptors at a higher ratio compared to NGF, and suggested that artemin has an important role on the regulation of TRP channels in primary afferents, as does NGF.

### Continuous artemin injection into the plantar surface caused mechanical and heat hyperalgesia

**Figure 5:**
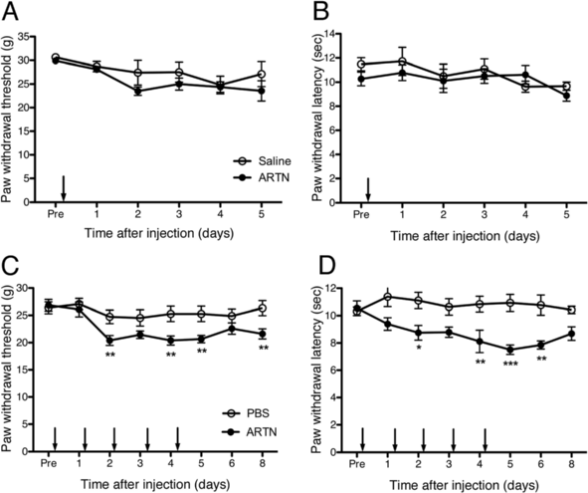
**Continuous artemin injection into the plantar surface caused mechanical and heat hyperalgesia. A**-**B**: Artemin (100 μg/ml, 100 μl/injection, PBS control n = 8) was injected into the planter surface only once and mechanical **(A)** and thermal **(B)** thresholds were tested after 5 days. A single dose of artemin, however, did not induce either mechanical or thermal hyperalgesia. **C**-**D**: Artemin (20 μg/ml, 100 μl/injection, PBS control, n = 8 each groups) was injected once a day for 5 consecutive days, resulting in significant changes in mechanical **(C)** and heat **(D)** thresholds, *p < 0.05; **p < 0.01; ***p < 0.001versus PBS group (two-way repeated ANOVA followed by Bonferroni post-test).

An analysis of expression of the ligands and receptors of artemin/NGF in primary afferent neurons indicated a physiological role in pain modulation. Although NGF has been believed to be one of the major contributing factors in inflammatory pain, the results of some previous studies suggested that artemin might cause hyperalgesia after peripheral inflammation. Therefore, in order to examine the role of artemin on pain behaviors, we injected recombinant mouse artemin (100 μg/ml, 100 μl) into the plantar surface once and measured mechanical and heat thresholds for 5 days. A single injection of artemin, however, did not induce hyperalgesia either in mechanical or in thermal testing (Figure 5A, B). Next, we examined the effects of repeated artemin administration into the plantar surface. We injected artemin (20 μg/ml, 100 μl/injection, PBS control, n = 8 for each group) once a day for 5 days and tested heat and mechanical thresholds (Figure 5C, D). Significant differences were found from the second day in both types of testing and they continued until the day following the final injection. These results suggested that the continuous generation of artemin might change gene expression in DRG neurons involved in hyperalgesia.

### Artemin increases TRPV1/A1 expression and Ca^2+^ activity in primary cultured DRG neurons

**Figure 6:**
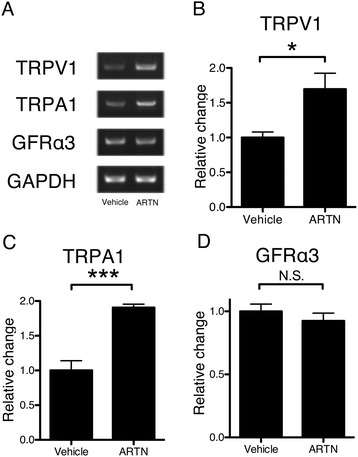
**Artemin increased the mRNA of TRPA1 and TRPV1 in primary cultured DRG neurons. A**: Artemin directly changed the gene expression of TRPV1 and TRPA1 in cultured DRG neurons using RT-PCR. **B**-**D**: Quantitative analysis showed a significant increase in mRNA levels of TRPV1 and TRPA1 after 18 hours of incubation with artemin (100 ng/ml), but not with GFRα3. (mean ± SEM, *p < 0.05, ***p < 0.001, compared with the vehicle group).

Using DRG primary culture neurons, we used RT-PCR to determine whether artemin obtained either from inflamed peripheral tissue or damaged nerves can directly change the gene expression of TRPV1 and TRPA1 in primary afferents (Figure 6A-D). Application of artemin into culture medium induced TRPA1 as well as TRPV1 (Figure 6A). There was a significant increase in mRNA levels of TRPV1 and TRPA1 after 18 hours of incubation with artemin (100 ng/ml), but not in those of GFRα3 (Figure 6B-D). These results confirmed that artemin directly regulates the gene expression of TRPV1 and TRPA1 in DRG neurons.

**Figure 7:**
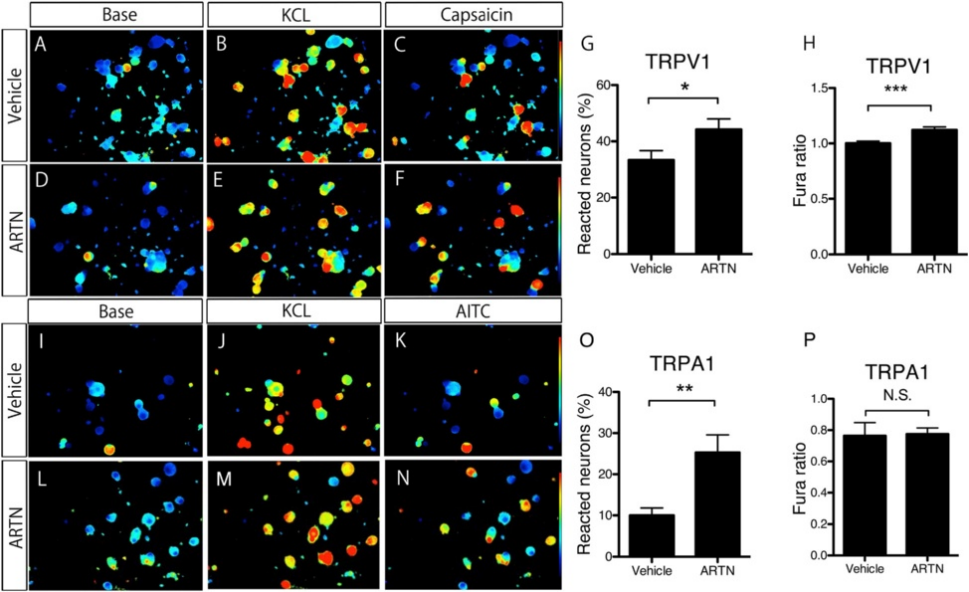
**Artemin increases Ca** ^**2+**^ **activity through TRPA1 and TRPV1 in primary cultured DRG neurons. A**-**F**, **I**-**N**: Examples of a Fura-2-based Ca2+ imaging method with DRG primary cultured neurons in vehicle- **(A-C, I-K)** or artemin-treated groups **(D-F, L-N)**. The images are before stimuli **(A, D, I, L)**, after KCL **(B, E, J, M)**, and after capsaicin **(C, F)** or AITC **(K, N)**. **G** and **O**: The percentage of TRPV1**-**(G) or TRPA1**-** (O) expressing neurons was significantly larger in artemin-treated neurons than amongst vehicle-treated ones. TRPV1 positive neurons were examined by application of capsaicin, and TRPA1-expressing neurons were confirmed by the application of AITC (100 μM) and capsaicin (1 μM). **H** and **P**: The increase in the Fura ratio (the ratio of 340/380 nm values) between the two treated groups was compared. The artemin-treated DRG neurons showed higher Fura ratios after application of capsaicin than the vehicle-treated ones (***p < 0.001, **H**). However, the changes in Fura ratios in AITC/capsaicin responsive neurons did not show a significant difference between the artemin and vehicle-treated groups **(P)**. (mean ± SEM, *p < 0.05, ***p < 0.001, compared with the vehicle group).

Next, to test whether the increase in TRPV1/A1 mRNA produced by artemin is physiologically relevant, we used a Fura-2-based Ca^2+^ imaging method with DRG primary cultured neurons, incubated in the same medium as in the previous study (Figure 7A-F, I-N). Firstly, cell viability was checked with a high-concentration solution of KCl. Cells activated by KCl were considered to be neurons. TRPV1-expressing neurons were confirmed by the application of capsaicin (200 nM). The number of capsaicin responsive neurons was significantly larger in artemin-treated neurons than amongst vehicle-treated neurons (Figure 7G). We also compared the increase in the Fura ratio (the ratio of 340/380 nm values) between the two treatment groups (Figure 7H), and found that the artemin-treated DRG neurons showed a higher Fura ratio than the vehicle-treated neurons. Next, we examined TRPA1-expressing neurons in the same DRG cultured neurons. TRPA1 positive neurons were confirmed by the application of AITC (100 μM) and capsaicin (1 μM). The number of AITC/capsaicin responsive neurons was larger amongst artemin-treated cultured neurons than vehicle-treated neurons (Figure 7O). However, the Fura ratio in responsive neurons did not show any difference between the two groups (Figure 7P).

### Differential regulation of TRPV1/A1 expression by artemin in primary afferents

**Figure 8:**
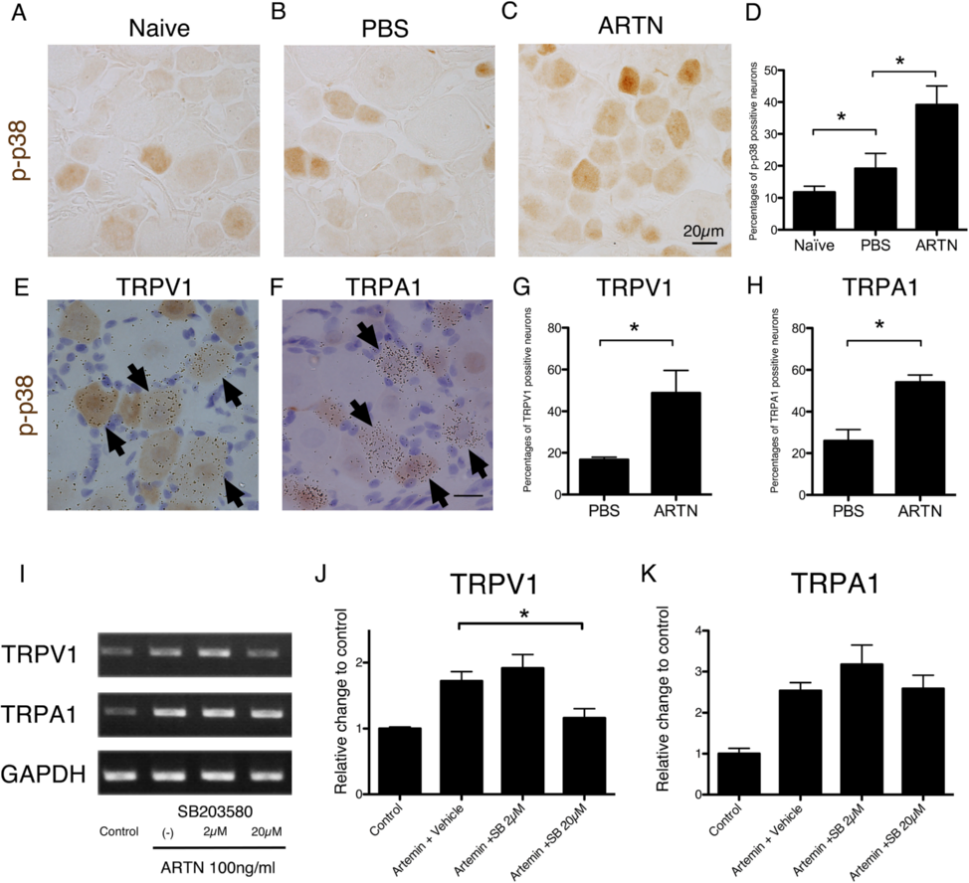
**p38 phosphorylation regulated TRPV1/A1 expression in DRG neurons. A**-**C**: Immunostaining of p-p38 positive DRG neurons in naïve and PBS- and artemin-injected rats. The repetitive plantar injection of artemin increased the number of p-p38 positive neurons compared to in the PBS control group. **D**: The percentages of p-p38-labeled neurons showed that repeated artemin injection increased p-p38-labeled neurons in the DRG compared to PBS control (mean ± SEM, *p < 0.05). **E** and **F**: Colocalization of TRPV1 or TRPA1 mRNA (clusters of black grains) and p-p38 (brown cells) using the double-labeling method with ISHH and immunohistochemistry. Arrows indicate examples of double-labeled neurons. **G**, **H**: The percentages of TRPV1- (G) or TRPA1 (H)-expressing neurons from amongst p-p38-labeled neurons. Colocalization of TRPV1 or TRPA1 in p-p38-labeled neurons was significantly increased after artemin injection (mean ± SEM, *p < 0.05). Bars, 20 μm **(A-C, E, F)**. **I**-**K**: RT-PCR of DRG primary cultured neurons with artemin (100 ng/ml) and two different doses of SB203580, 2 μM and 20 μM. The inhibition of mRNA was seen only with 20 μM of SB203580 for TRPV1 and not observed for TRPA1 (mean ± SEM, *p < 0.05; versus artemin + vehicle group).

Previous studies have reported that phosphorylation of p38 MAPK is involved in the regulation of TRPV1 mRNA expression in DRG neurons [26,27]. To test if the increase of TRPV1 and TRPA1 expressions in DRG neurons by artemin is mediated by the p38 MAPK pathway, we performed immunohistochemistry using a phospho-p38 antibody (activated p-38 antibody) (Figure 8A-C). Previous studies reported that p-p38 is present only in 15% of DRG neurons in naive rats [26]. Although repeated PBS plantar injection increased the number of p-p38-positive neurons compared to naive neurons, repeated artemin plantar injection (2 μg) increased them to a greater degree than PBS control neurons (Figure 8D). The difference between the PBS injected group and artemin injected group was statistically significant. Furthermore, we performed double-labeling experiments for TRPV1 or TRPA1 mRNA and p-p38 by a method combining ISHH and immunohistochemistry (Figure 8E, F). A number of TRPV1 or TRPA1 mRNA expressing neurons in the artemin injection group were also labeled with p-p38 MAPK. The percentages of colocalization of TRPV1 or TRPA1 in p-p38-labeled neurons were significantly increased after artemin injection (Figure 8G, H). Therefore, these results led us to examine a possible relationship between the increases in TRPV1 and TRPA1 mRNA in DRG neurons and the p-38 MAPK pathway.

Based on our previous results, we hypothesized that increases in TRPV1 and TRPA1 produced by artemin are mediated through the p38 MAPK pathway. To test this, we examined the effect of SB203580 on the expression of TRPV1/A1 using RT-PCR of primary cultured DRG neurons (Figure 8I-K). We co-administrated SB203580, 2 μM or 20 μM and artemin simultaneously and incubated the cells for 18 hours. Twenty μM of SB203580 significantly reversed the increase of TRPV1 mRNA (Figure 8I, J), however SB203580 had no effect on the artemin-induced TRPA1 upregulation (Figure 8I, K). It suggested that p38 MAPK is involved in the regulation of TRPV1 upregulation by artemin, but not of TRPA1.

## Discussion

In recent years, there has been interest in the study of artemin, a GDNF family member, because it has shown a very wide variety of effects, including bi-directional results in the modulation in neuropathic and inflammatory pain. Previous studies suggested a possible role of artemin as a survival factor of sensory and sympathetic neurons *in vitro* and *in vivo.* Systemic or intrathecal administration of artemin was shown to prevent the alteration of the nervous system that occurs after peripheral nerve injury [19-22]. Nerve injury-induced pain behaviors were also dose-dependently reversed by these treatments [20]. In contrast, artemin is known to have a pivotal role in pain hypersensitivity after peripheral inflammation or nerve injury. For example, the important paper by Elitt et al. [2] reported that the overexpression of artemin increased the expression of TRPV1 and TRPA1 in primary afferents and subsequent hyperalgesia, and increased neuronal activity. The application of artemin induced a significant potentiation of TRPV1 function *in vitro* and thermal hyperalgesia [24]. These bi-directional effects have been reported with another well-known neurotrophic factor, nerve growth factor (NGF). NGF is known to have an important role in the positive regulation of many pronociceptive molecules in primary afferents and also to increase pain behaviors. However, NGF has been found to reverse hyperalgesia in a neuropathic pain model when it was applied to a nerve injury site [28,29]. Bi-directional effects of artemin in persistent pain models clearly require further study.

Another interesting point is that artemin may have different effects on TRPV1 and TRPA1. We reported that short-term application of artemin inhibits TRPA1 channel activity and subsequent pain behaviors [25]. However, acute application of artemin was reported to induce potentiation of TRPV1 and produce acute thermal hyperalgesia [24]. Overall, these findings suggest that acute administration changes the expression of TRPA1 and TRPV1 differentially, and also that acute and long-term exposure to artemin has different effects on the activity of TRP channels. Therefore, the purpose of this study was to examine the effects of long-term or repeated application of artemin on pain behaviors and on the expression of TRP family channels using *in vivo* and cultured DRG neurons. In the behavioral analysis in the present study, we confirmed that the repeated or continuous injection of artemin into the rat plantar surface induced mechanical and heat hyperalgesia. This is consistent with previous papers, in which artemin was injected peripherally into the mouse hindpaw [24,30], and anti-artemin partially reversed CFA-induced mechanical hyperalgesia [31].

In the present study, we found that artemin mRNA was increased in the epidermis after CFA injection. Previous papers showed an artemin increase in the skin after CFA-induced peripheral inflammation [24,30] but lacked a morphological analysis of the skin. The cells expressing increased artemin mRNA have not been precisely identified but is presumed that they were keratinocytes. Artemin overexpression in mice, where the artemin gene was inserted after the K14 keratin promoter, produced a similar pattern of phenotypic changes of TRP channel expression in primary afferents, as well as behavioral changes, as found in the present study [2,23]. The *in situ* hybridization signals showing artemin mRNA apparently increased in the epidermis 1d after CFA injection (Figure 1O, R) suggesting that keratinocytes express increased artemin mRNA. A paper examining human atopic dermatitis patients’ skin showed that fibroblasts, which accumulated in the lesion, expressed increased artemin [32]. Therefore, the precise cellular origin of increased artemin in the inflamed epidermis/dermis is not clear at this point. In the dermis or hypodermis, there were a number of cells showing intensified signals of artemin mRNA after CFA injection. Eosin-labeled immune cells in the dermis also expressed increased artemin mRNA (Figure 1S); however, the detailed classification of the cells was not determined due to methodological limitations. In addition, smooth muscle-like cells in the blood vessels in the dermis expressed increased NGF mRNA after CFA injection (Figure 1K). An interesting point on NGF/artemin expression in the skin after CFA-induced inflammation is that it was much longer than that of NGF; the NGF increase terminated 1d after CFA injection. In contrast, the artemin increase started at 1d and continued for a few days. The long-term effects of artemin on pain may be more important than those of NGF, at least in the skin.

In the present study we found that artemin mRNA increased in the sciatic nerve distal to the nerve injury site. The sciatic nerve tissue was examined by RT-PCR, and *in situ* hybridization did not include the injury site, indicating the changes observed in the present study may not be a direct result of changes at the nerve injury site but may involve changes in the distal nerve, such as via Wallerian degeneration. Compared to NGF, artemin mRNA showed large and long-lasting upregulation after nerve injury. The increase was first detectable 7d after injury and continued for at least 3 weeks. These data also indicate that artemin has the characteristics of a long-lasting trophic factor in primary afferents.

In terms of regulatory effects of artemin on gene expression in primary afferents, most studies suggested a positive role of artemin on TRP channel expression [23,24,32]. For example, TRPA1/V1 increases with artemin overexpression in mice and artemin and TRPA1/V1 increases *in situ* in inflamed tissue. In the present study, we confirmed the direct regulatory effect of artemin on TRPA1/V1 expression in the primary afferent in two ways. Firstly, mRNA analysis of DRG tissue was done by RT-PCR and *in situ* hybridization after artemin injection into the rat hindpaw *in vivo* (Figure 3). Secondly, TRPA1/V1 mRNAs were measured using cultured rat DRG neurons (Figure 6). We could clearly show that artemin administration significantly up-regulated the expression of TRPA1/V1 in small to medium-sized DRG neurons, and we believe that these data are the first demonstration of direct effects of artemin on TRP channel expression in DRG neurons.

The time-course of the up-regulation of artemin and NGF after peripheral inflammation by CFA is intriguing (Figure 1). NGF produced a rapid and transient increase in mRNA, in contrast to artemin which had a more robust and long-lasting upregulation in the skin after CFA injection. This is consistent with data from mouse skin after CFA injection [24]. Also the double-labeling experiments in the present study (Figure 4) indicate a higher colocalization between TRPA1/V1 and the artemin receptor, GFRα3, compared to that between TRPA1/V1 and the NGF receptor, TrkA. These data also support the pivotal role of periphery-derived artemin as a regulator of TRP channels in primary afferents. Considering that the injection of the rat plantar surface with artemin induces a significant increase of TRPV1/A1 expression in small to medium-sized L4-5 DRG neurons, the regulatory role of artemin on TRP channel expression and nociception is likely to be distinct and potent, like with NGF.

The p38 MAPK pathway has an important modulatory role of TRPV1 [26,27,33] and TRPA1 [34-36]. Ji et al., [26] found that activation of p38 in the DRG by periphery-derived NGF increases TRPV1 levels in nociceptor peripheral terminals and contributes to the maintenance of inflammatory pain. Therefore, we examined whether artemin regulates TRP channels in the DRG through the p38 MAPK pathway, and found the up-regulation of p-p38 and colocalization of p-p38 and TRPA1/V1 in the DRG *in vivo* after artemin treatment. However, we demonstrated that the p38 inhibitor SB203580 significantly suppressed artemin-induced upregulation of TRPV1 in a DRG culture, and did not affect on TRPA1 expression. As previous papers suggested that p38 activation in small to medium-sized DRG neurons might have a very important role in the expression of TRPV1 or other pain related molecules [26,27,33], the present finding that artemin-induced p38 has a pivotal role in TRPV1 channel expression is not surprising. Our data suggest that TRPA1 upregulation by peripherally increased artemin is mediated through different signaling cascades from p38 MAPK pathway, and the precise mechanism how artemin regulates TRPA1 expression in primary afferent neurons should be clarified. NGF has been long recognized as the only regulator of pain-related molecules in nociceptive neurons of primary afferents. However, we found increased artemin synthesis in the peripheral tissue after inflammation or nerve injury, which may have an important role in the regulation of TRP channel expression, as is the case with NGF.

## Conclusion

We have demonstrated that artemin increases locally in skin over long periods of time after peripheral inflammation and in the sciatic nerve following Wallerian degeneration after nerve injury. Repeated artemin injections into the periphery changed the gene expression of TRPA1/V1 and TRPA1 in DRG neurons and induced mechanical and heat hyperalgesia. Our data using primary cultured DRG neurons suggest that TRPV1 upregulation by artemin is mediated through p38 MAPK, however artemin-induced TRPA1 increase may have a different signaling pathway. All findings here indicate the important role of peripherally-derived artemin on the regulation of TRPV1/A1 in DRG neurons.

## Methods

### Animals

All animal experiments conformed to the regulations of the Hyogo College of Medicine Committee on Animal Research and were performed in accordance with the guidelines of the National Institutes of Health on animal care. Male Sprague Dawley rats, aged 4 weeks for the DRG primary culture neurons and calcium imaging study, and those weighing 200–250 g for the other studies, were used. Plantar injection was performed under ether anesthesia, and surgical procedures were performed under sodium pentobarbital (50 mg/kg, i.p.) anesthesia. Additional doses of the anesthetics were given as needed. 50% Complete Freund’s adjuvant saline (CFA, 100 μl) was injected into the plantar surface of the left hind paw. Recombinant mouse artemin (R & D systems, 20 μg/ml, 100 μl) was injected into the plantar surface. DMSO was used as a vehicle control for the models using p38 inhibitor with the DRG primary culture neurons. L5 spinal nerve ligation was performed with some modifications to the original SPNL model [37]. After anesthetizing the rats, the hair of the lower back was shaved and the skin was sterilized with 0.5% chlorhexidine. Using sterilized operating instruments, the left L5 spinal nerve was isolated and tightly ligated with 3–0 silk thread (L5 SPNL). Thereafter, the ligated spinal nerve was cut on the distal side to the ligated part. The right side was not subjected to any surgery. The wound was washed with distilled saline and sutured with 3–0 silk thread.

### *In situ* hybridization histochemistry

Rats were perfused transcardially with 1% paraformaldehyde in 0.1 M phosphate buffer (PB, pH 7.4) followed by 4% paraformaldehyde in 0.1 M PB. The bilateral L4-5 DRG neurons and sciatic nerve (1 cm length) 2 cm distal to the ligation were removed, and postfixed in the same fixative at 4°C overnight, followed by immersion in 20% sucrose in 0.1 m phosphate buffer at 4°C for 2 d. Thereafter, they were rapidly frozen in powdered dry ice, and cut on a cryostat at a thickness of 8 μm. For plantar skin samples, rats were sacrificed by decapitation under deep ether anesthesia. The plantar skin surface was removed, rapidly frozen, and cut on a cryostat at a thickness of 5 μm. Sections were thaw-mounted onto MAS-coated glass slides (Matsunami, Osaka, Japan). The details of the dual ISHH procedure have been described in our previous study [6]. For autoradiography, the sections were coated with NTB emulsion (Eastman Kodak, Rochester, NY) diluted 3:2 with distilled water at 45°C and exposed for 1–2 weeks in light-tight boxes at 4°C. After development in D-19 (Eastman Kodak) and fixation in 24% sodium thiosulfate, the sections were stained with hematoxylin-eosin, dehydrated in a graded ethanol series, cleared in xylene, and coverslipped [6].

For the co-expression study of the two different mRNAs on DRG neurons in naive rats, we used ^35^S-labeled RNA probes for TRPV1 (GenBank accession number AF029310, nucleotides 149–505), TRPA1 (GenBank accession number AY496961, nucleotides 302–788), and DIG-labeled probes for TrkA (GenBank accession number X05137, nucleotides 560–979) and GFRα3 (GenBank accession number AF184920, nucleotides 164–604) in the same sections.

### Immunohistochemistry

The protocol for immunohistochemistry was based on the published ABC (Vectastain Elite ABC kit, Vector, CA, USA) method [38]. The DRG sections on the MAS-coated glass were preincubated in TBS containing 10% normal goat serum (NGS) for 1 hour, then incubated in primary antibody for p-p38 (1:500, Cell Signaling Technology) in the same solution for 48 hours at 4°C. The sections were washed in TBS and then incubated in biotinylated anti-rabbit IgG (1:200; Vector Laboratories, Burlingame, CA) in TBS containing 5% NGS for 2 hours at 4°C, followed by incubation in avidin-biotin–peroxidase complex (Vectastain Elite ABC kit, Vector, CA, USA) for 1 hour at room temperature. The horseradish peroxidase reaction was developed in 0.1 M Tris-buffered saline, pH 7.4, containing 0.05% 3,39-diaminobenzidine tetrahydrochloride (Sigma, Steinheim, Germany), and 0.01% hydrogen peroxidase. Sections were then washed in TBS, mounted on slides, dried, and cover-slipped.

### Image analysis

The statistical analyses of ISHH have been previously described in detail [38-40]. At least 3,000 neuronal profiles from four rats were quantified for each antisense probe in the single ISHH study. Measurements of the density of silver grains over randomly selected tissue profiles were performed using a computerized image analysis system (NIH Image, version 1.61), in which only neuronal profiles that contained nuclei were used for quantification. At a magnification of 200x and with brightfield illumination, upper and lower thresholds of gray level density were set such that only silver grains were accurately discriminated from the background in the outlined cell or tissue profile and were read by the computer, pixel by pixel. Subsequently, the area of discriminated pixels was measured and divided by the area of the outlined neuronal profile, giving a grain density (%) for each cell. To reduce the risk of biased sampling of the data because of varying emulsion thickness, we used a signal-to-noise (S/N) ratio for each cell in each tissue. The S/N ratio of an individual neuron and its cross-sectioned area, which was computed from the outlined profile, was plotted. Based on this scattergram, neurons with a grain density 5-fold that of the background level or higher (5 S/N ratio) were considered positively labeled for TRPA1, TRPV1 and GFRα3 mRNAs. To distinguish cell size-specific changes, we characterized the DRG neurons as small (<600 μm^2^), medium (600-1200 μm^2^) and large (>1200 μm^2^), according to their cross-sectional area. Since a stereological approach was not used in this study, quantification of the data may represent a biased estimate of the actual numbers of neurons. The number of positively labeled DRG neurons was divided by the number of neuronal profiles counted in each DRG. Data are expressed throughout as mean ± SEM (%). The level of statistically significant difference was taken to be a probability of less than 5% (p < 0.05) [40].

### Reverse transcription-polymerase chain reaction (RT-PCR)

For the CFA plantar injection model, the rats were sacrificed by decapitation under deep ether anesthesia at 3 h, 9 h, 12 h, 1d, 3d and 7d after CFA injection (n = 4 at each time point). The plantar skin was removed bilaterally. The SNL model rats were sacrificed by decapitation under deep ether anesthesia at 1d, 3d, 5d, 7d, 14d and 21d after the operation (n = 4 at each time point). The bilateral 1 cm long sciatic nerves were removed 2 cm away from the ligation. The samples were removed and rapidly frozen with powdered dry ice. They were stored at −80°C until use. Skin samples were cut on a cryostat at 40 μm thickness and collected in a 2.0 ml collection tube. Extraction of total RNA was carried out using the RNA extraction reagent ISOGEN (Nippon Gene, Tokyo, Japan). For the DRG primary culture neurons, the incubated culture was washed with TBS and collected with 1 ml of ISOGEN. All samples were homogenized in 1 ml of ISOGEN regent, mixed with 200 μl of chloroform, and centrifuged for 15 minutes at 4°C and 12,000 *g*. The supernatant was mixed with 500 μl of isopropyl alcohol and centrifuged again under the same conditions.

For purification of total RNA from these samples, a PureLink^®^ RNA Micro Kit (Invitrogen, CA, USA) was used. RNA extraction was quantified by measurement of optical density at 260 nm. Samples of 3 μg of total RNA were mixed with 25 μl RT mixture to a final concentration 200 units of M-MLV reverse transcriptase (Promega, Madison, WI), 20 units of RNase inhibitor (Promega), 0.8 μM dNTP, and 1 μg oligo dT primer in 1 x reaction buffer (pH 7.5; Promega). RT was carried out at 37°C for 60 minutes, following inactivation at 70°C for 10 minutes. The forward and reverse primers specific for rat NGF, artemin, TRPV1, TRPA1, GFRα3 and GAPDH were designed as shown in Table 1. The PCR reaction was performed in 10 μl of Taq PCR master mix kit (QIAGEN, CA, USA) and cDNA with a pair of 10-pmol primers for each gene on a DNA Thermal Cycler (GeneAmp PCR System 9700, Applied Biosystems, Foster City, CA). The PCR conditions for temperature cycles were different for each gene. The resulting PCR products were electrophoresed through a 1.25% agarose gel containing ethidium bromide and visualized with UV illumination. Each pair of forward and reverse primers presented a single band with the expected size.

**Table 1:** **Sequence locations of primers used in this study**

**Gene**	**GenBank accession no.**	**Primer**	
		**Forward**	**Reverse**
NGF	M36589	652-671	1016-997
Artemin	NM053397	852-871	1252-1233
TRPV1	AF029310	149-168	505-486
TRPA1	AY496961	302-321	788-769
GFRα3	AF184920	164-183	604-585
GAPDH	M17701	80-99	350-331

### Behavioral studies

Adult male Sprague–Dawley rats (200-250 g) were used for the behavioral analyses. Room temperature and lightning were kept stable for all testing. Rats were sufficiently habituated in the environment and tested for basal pain hypersensitivity. Thereafter, rats were administered 100 μl of artemin or PBS into the left hindpaw. One day after the administration, mechanical and thermal behavioral tests were started with a Dynamic Plantar Aesthesiometer (Ugo Basile, Italy) and the Hargreaves radiant heat apparatus, respectively. The Dynamic Plantar Aesthesiometer was used as the automated von Frey hair system. The threshold was taken as the force applied to the plantar surface at which the rats withdrew from it. For the 5-consecutive-day injection model, testing was performed just before injection. The first value for each test was removed from the data, and the averages from the subsequent three values were taken as the thermal and mechanical thresholds.

### DRG primary culture neurons

DRG neurons were removed from 4-week Sprague–Dawley rats with a sterile technique, and were put into ice-cold Earle’s balanced salt solution (EBSS, Sigma). In order to remove the adhered fat and connective tissue, the DRG neurons were placed in 2 ml of EBSS with 1.25 mg/ml of collagenase P (Sigma) for 2 hours at 37°C. After incubation, the cell suspension was centrifuged for 5 minutes at 1000 rpm and the cell pellet was re-suspended in EBSS. The DRG neurons were mechanically dissociated with a Pasteur pipette in Neurobasal medium, 10% FCS, D-MEM, 2% B27 supplement plus (Miltenyi Biotec Inc, CA, USA), SMPCG and L-Glu. After dissociation, the cell suspension was filtered, plated onto poly-L-lysine coated plates, and incubated with the medium for 2 days. In order to exclude the effect of other growth factors, Neurobasal with 2% of B27 supplement plus, SMPCG and L-glu were used. For the artemin group, recombinant mouse artemin (100 ng/ml) was added to the medium. In the experiment with p38 MAPK inhibitor, SB203580 [4-(4-fluorophenyl)-2-(4-methylsulfinylphenyl)-5-(4-pyridyl)-1*H*-imidazole] (Calbiochem, La Jolla, CA, USA) dissolved with DMSO was administered to the medium with artemin at the same time. SB203580 were used with two different dose: 2 μM and 20 μM. After an 18-hour incubation period, the DRG neurons were removed with a cell scraper and put into 1 ml of ISOGEN (Nippon Gene, Tokyo, Japan).

### Ca^2+^ imaging study

Ratiometric calcium imaging was performed using an Olympus fluorescence microscope equipped with a variable filter wheel (Sutter Instruments, Novato, CA, USA) and a cooled digital CCD camera (Hamamatsu, Shizuoka, Japan). Dual images (340 and 380 nm excitation, 510 nm emission) were collected and pseudo-color ratiometric images were monitored every 2 seconds during the experiment using an Axon Imaging Workbench 5.0 (INDEC BioSystems, Inc, Mountain View, CA, USA) [41]. The DRG neurons were removed from 4-week Sprague–Dawley rats. After dissociation of the DRG neurons, the cell suspension was placed onto coverslips coated with poly-L-lysine and cultured for 18 to 24 hours with the same method as above (see DRG primary culture neurons in the Methods section). They were loaded with 4μM Fura-2 acetoxymethyl ester (Nacalai Tesque, Kyoto, Japan) for 40 minutes at 37°C. After the incubation, the coverslips were placed in a chamber connected to a gravity flow system to deliver various chemicals. One microscopic field on the coverslip, which contained 20 to 30 neurons, was randomly selected and measured. The data were collected from a total of 20 fields in each group (n = 4 each group). The system was loaded with a standard bath solution containing 140 mM NaCl, 5 mM KCl, 2 mM MgCl_2_, 2 mM CaCl_2_, 10 mM HEPES and 10 mM glucose pH7.4 (adjusted with NaOH). A standard bath solution was first applied to the cells for 20 seconds, followed by KCl (50 mM) for 20 seconds, and the cells were then washed with the standard bath solution for 5 minutes. For the TRPV1 study, capsaicin (200nM) was applied for 20 seconds and it was washed out again with the standard bath solution for 3 minutes. For the TRPA1 study, AITC (100 μM) was applied for 20 seconds and it was washed out for 3 minutes. Thereafter, capsaicin (1 μM) was perfused for 20 seconds. The number of agonist-responding neurons relative to KCl-responding neurons in each selected microscopic field was calculated and summed.

### Quantification and Statistics

All results are expressed as mean ± SEM. An unpaired *t*-test was used for RT-PCR of DRG primary culture neurons and the calcium imaging data to compare the groups. A one-way ANOVA was used for the RT-PCR of CFA plantar injected skin and DRG primary culture with the p-p38 inhibitor. For all behavioral testing, a two-way ANOVA followed by Bonferroni posttests was used. Differences were accepted as significant if the probability was less than 5% (p < 0.05). All emulsion-coated slides were digitized with a Nikon DIAPHOT-300 microscope connected to a Nikon DXM1200 digital camera. We used Adobe Photoshop Element 10.0 (Adobe Systems, Mountain View, CA, USA) to optimize the images and prepare all figures.
